# Challenges of accessing hygiene facilities when on the move: an exploratory interview study with UK mobile workers

**DOI:** 10.1186/s12889-023-17465-y

**Published:** 2023-12-15

**Authors:** Sophie Rutter, Andrew Madden, Lauren White

**Affiliations:** 1 Information School, The Wave, 2 Witham Road, S10 2AH Sheffield, UK; 2 Sheffield Methods Institute, The Wave, 2 Witham Road, S10 2AH Sheffield, UK

**Keywords:** Hand hygiene, Toilets, Mobile workers, Inequalities, Workplaces

## Abstract

**Background:**

Access to hygiene facilities is essential for health and well-being, and in many countries, employers are legally obliged to ensure that hygiene facilities are readily available. This interview study considers how being on the move impacts the ability of mobile workers (such as community care workers, police, delivery drivers, gardeners, cleaners, utility workers) to access hygiene facilities, and the challenges they face.

**Methods:**

Using a qualitative exploratory research design, we investigate through semi-structured interviews with 22 United Kingdom (UK) mobile workers (1) what influences their access to hygiene facilities, (2) their hygiene needs, and (3) where mobile workers are accessing hygiene facilities. The interview data was analysed qualitatively using a coding framework developed from a literature review of hand hygiene in fixed workplaces.

**Results:**

Mobile workers’ access to hygiene facilities is influenced by the wider cultural environment, the biological environment, the organisational environment, the physical environment, the facility owner, the worker’s role, and the individual themselves, all underpinned by social norms. Our participants needed hygiene facilities so they could use the toilet, clean themselves, and do their work, and for First Aid. Access to facilities is challenging, and our participants needed to access facilities where they were working, travel to find them, or use hygiene kits. The quality of facilities is frequently poor, and mobile workers must often seek permission and may incur financial costs. Our participants often had to rely on the goodwill of people in private homes. In the absence of facilities, workers often resort to strategies that may affect their health (such as restricting drinking and eating, and ignoring urges) or their dignity (such as relieving themselves outdoors or even soiling their clothes).

**Conclusions:**

The lack of hygiene facilities available to mobile workers is a serious health and well-being concern. Given that there are many occupations where workers are mobile at least some of the time, the scale of the problem needs to be recognised. This study adds to our understanding of hygiene in workplaces and highlights the inadequacy of current legislation, which appears to serve primarily those working in fixed workplaces such as offices. Recommendations are made to policy makers and organisations.

**Supplementary Information:**

The online version contains supplementary material available at 10.1186/s12889-023-17465-y.

## Background

Providing opportunities to access hygiene facilities in workplaces is necessary for health and wellbeing [1] and is a significant occupational health issue [2]. Good hygiene protects workers from acquiring and spreading infections [3], and access to hygiene facilities prevents health issues associated with restriction of fluid intake [4] and / or the ignoring of urges [5]. Providing access is also a health equalities issues as the impact varies on different groups of workers: for example, workers who menstruate and those with protected characteristics such as older workers, disabled workers, and those with health conditions are particularly affected [2, 6–8]. The importance of hygiene is widely recognised in workplace regulations (see for example, regulations from the UK Health and Safety Executive [9]) whereby employers are required to provide hygiene facilities. However, depending on the worker’s occupation and background access to facilities can still be challenging [2]. This exploratory study considers how being on the move and away from a fixed work base impacts on mobile workers’ access to hygiene facilities. This is important as there has been little research in this area, yet the health impacts on mobile workers can be considerable.

Mobile workers spend most of their time away from a work base [10] because either movement is essential to the work (e.g. delivery drivers, food couriers) or because their work is geographically displaced (e.g. plumbers, carers). Business travellers who work on the move while away from fixed locations are also mobile workers [11]. Occupational challenges for mobile workers include feeling isolated, long hours, and reduced access to resources [12].

Studies on the health and well-being of mobile workers have focused on the demands of the job, for example, for international business travellers [13], and for construction workers and repair engineers [14]. A further stream of research has considered the musculoskeletal issues associated with being in vehicles [10]. Recent studies have also considered the health implications of working in places far away from home [15] and the risks of catching COVID-19 while commuting [16]. Lorry drivers’ and truckers’ limited access to sanitation facilities has also been highlighted [17, 18]. However, there has been little consideration more generally, of how being on the move and away from a fixed work base impacts on workers’ access to hygiene facilities. For this study, we recruited mobile workers from different occupations to discuss access to hygiene facilities while they are at work. Our research questions were as follows:

- RQ1: What is influencing mobile workers access to hygiene?
- RQ2: What are the hygiene needs of mobile workers?
- RQ3: Where are mobile workers accessing hygiene facilities?

### The requirement for hygiene facilities at work

The value of good hygiene in the workplace is sufficiently recognised that, in many countries, employers are legally obliged to ensure that hygiene facilities are readily available. In the UK for example, “Sufficient toilet and washing facilities should be provided to allow everyone at work to use them without unreasonable delay” [9]. However, despite this recognition and legislation, not all workers are able to access hygiene facilities. In a 2010 campaign, the UK’s Trades Union Congress (TUC) complained that “many employers… plan work which takes no account of toilet breaks or develop a work culture where use of the toilet whenever it is required is frowned on” and for some workers “there are no toilet facilities provided or they are closed at certain times, such as at night” [19].

The Health and Safety Executive (HSE) [20] advises employers that they must provide hygiene facilities where possible (portable toilets, chemical toilets and water containers if necessary) and that “public toilets and washing facilities should be a last resort”. Mobile workers must also be allowed to access the hygiene facilities of the workplace they visit. However, many workplaces have not provided this access and so the Health and Safety Executive are currently needing to update their guidance [21]. Furthermore, many mobile workers may visit private homes where there is no obligation to provide access to hygiene facilities, or places (such as parks) where there are no hygiene facilities.

The COVID-19 pandemic has given particular prominence to workplace hygiene. For example, in 2020 the UK government issued hygiene advice for “working safely during COVID-19 in offices and contact centres” [22], including where to place sanitisers and on the cleaning of toilets. However, not all workers have had such consideration, and unions have been campaigning for their members to be supplied with personal protection equipment (PPE) at work [23]. The plight of workers such as delivery drivers trying to gain access to hygiene facilities during lockdown has been well documented [24]. The lack of toilet facilities was highlighted in a 2019 report by the Royal Society for Public Health, which detailed the extent to which public toilets are disappearing from towns and cities in the UK. As a result, “Workers ‘on the move’, such as tourist guides, drivers of lorries or buses and postal workers, have often complained in vain of the lack of facilities” [25].

To gain a deeper understanding of issues that could be affecting mobile workers’ access to hygiene facilities we review two streams of research. The first considers more generally, who is and who is not able to access public toilet facilities. The second considers what influences hand hygiene in workplace settings.

### Public toilet access

Access to toilets is a universal, yet infrequently discussed, necessity of our everyday lives. However, questions of access and attention to such bodily requirements are not felt evenly across social groups. While globally there is increased recognition of the need to provide sanitation infrastructure in public institutions and places [26, 27], the pursuit of equitable provision and inclusion for *all* still remains. Access to toilets has been, and continues to be, shaped by place [28, 29], gender [30], race [31], age [32], class [24], disability [33], health conditions [34] and menstruation [35]. The time it takes to reach facilities, and whether (and for whom) they are open, also varies [36]. Inadequate access to toilets has implications for health and wellbeing, such that the ability to go about daily life and work (and significantly, the ability to be *mobile*) is constrained by lack of public toilet access.

The wealth of literature on toilet access from across social science disciplines and public health reveals the significant inequalities and impacts for the health and wellbeing of individuals and society. Access to toilets for *workers* is a significant, but less documented, part of this.

### Hygiene in workplace settings

Although mobile workers will likely be accessing hygiene facilities for more reasons than hand hygiene, prior studies on what influences hygiene behaviour in workplaces provides a promising basis to consider more generally what influences mobile workers’ access to hygiene facilities (see Supplementary Material for a summary of each study reviewed). Much of the research in this area is based in healthcare settings. To capture a diversity of influences, we interpret workplace settings broadly, and include education settings and the perspectives of informal carers in domestic settings.

Not surprisingly, studies have found that access is influenced by the physical environment, including where facilities are located, whether they are accessible and visible, if there is a cost to use them, how much privacy there is, whether hygiene materials such as soap can be accessed, and the quality of facilities and hygiene materials [37–47]. However, hygiene in the workplace is influenced by more than the physical environment. It is also influenced by the individual worker, the worker’s role, the organisational environment, the biological environment, the wider cultural environment and social norms.

An individual’s knowledge, behavioural capacity, motivation, habits and planning are important. Workers need to know when, how and why they should clean their hands; they must have the skill and the motivation to do so; furthermore hygiene is planned and habitual [37–40, 43–48].

The worker’s role is also important. Given constraints imposed by workload, workflows and work procedures, a worker may perceive there is insufficient time [38, 42, 45, 47]. This can depend on their professional status (e.g. nurse, doctor); associated work tasks and any competing priorities [38, 42, 46] as well as the hours they are working [46].

Related to the worker’s role is the organisational environment. The work domain (e.g. health, education) and organisational culture; the structure and ownership of the organisation (public, private); the policies, regulations, rules & encouragement; leadership; as well as whether the organisation provides any training and education [37, 38, 41–43, 45, 46, 49].

The biological environment affects hygiene. The visibility and presence of disease, faeces and urine all increase motivation [40]. The wider environment is also influential as socio-political factors and culture affect policies and attitudes [39, 40, 44, 49]. Finally social norms (i.e. prevailing behaviour) underpin all the factors identified (i.e. the physical environment, the individual, the workers’ role, the organisational environment, the biological environment and the wider cultural environment) [38–40].

The review above of what influences access to hygiene facilities in workplaces indicates that there are multiple competing and intersecting factors. The influence of these factors will vary for each workplace setting. Furthermore, what is important in one setting may not be important in another. What has not been considered, is what might be influencing access to hygiene facilities when workers are away from a fixed work base.

## Methods

An exploratory semi-structured interview study was conducted to identify what is influencing access to hygiene (RQ1), the hygiene needs of mobile workers (RQ2), and, where mobile workers are accessing facilities (RQ3). During the interviews, we encouraged UK mobile workers to share their experiences of accessing and using hygiene facilities during their working day. To give interviewees maximum opportunity to lead the conversation. all interviews began with an open question “*What’s a typical day like for you*?” This gave us insight into how our research aims mapped onto the interests, experiences and concerns of the participant, allowing us to adapt subsequent questions to ensure the relevance to their situation. Interviews were either by video link or by telephone, and took place between March and July 2022.

Care was taken to ensure that participants gave their informed consent, and we adopted an ethics in practice approach by ensuring that verbal consent and agreement continued throughout all stages of the research. Prior to taking part in the study, participants were given an information sheet that informed them of the nature of the research, its aims, what the process involves, and its anticipated outcomes. This information was revisited at the interviews. Participants were given opportunities to ask questions and were advised that they could withdraw with no negative consequences. Interviews were audio recorded and transcribed after each interview. To ensure confidentiality the transcripts were anonymised and references to any persons and commercial settings were removed. Consequently, all quotes are reported anonymously in this paper. To enable readers to place the quote in context, the quoted worker’s occupation is indicated. All interviewees were compensated for their time with a £25 high street shopping voucher.

Most of the interviewees were recruited by snowball sampling [50], but some responded to adverts posted on Twitter or LinkedIn, and distributed printed flyers. In all, 19 interviews took place, but on three occasions, two people attended the interview together at their request. In one case, the workers worked alongside each other, in another they shared a workplace but held different positions, and in the third, they had distinct jobs but shared a home. A total of 22 people from the UK were therefore engaged in discussions about their experiences of hygiene at work. We recruited participants by asking “do you work on the move or away from a central base”, thereby letting participants decide if they identified as a mobile worker. There were no other explicit inclusion / exclusion criteria. Some mobile workers had more than one job and/or had previous experience of a different job where they were also mobile. We responded to this flexibility within the open-ended nature of the interviews. This approach, led to us recruiting participants with experience of a diverse range of occupations including cleaners (1), community support workers (1), community care workers (4), construction workers (1), delivery drivers (4), freelancers who moved between workplaces (5), gardeners (2), the military (3), park rangers (1), the police (3), the post office (1), teachers (1), utility workers (1), and window cleaners (1). In the results we attribute quotes to the occupation the mobile worker was referring to at that time. Where the occupation was represented by more than one interviewee we distinguish contributors with a number based on the order in which we interviewed them (e.g. community care worker #2 was the second community care worker to be interviewed).

**Fig. 1:**
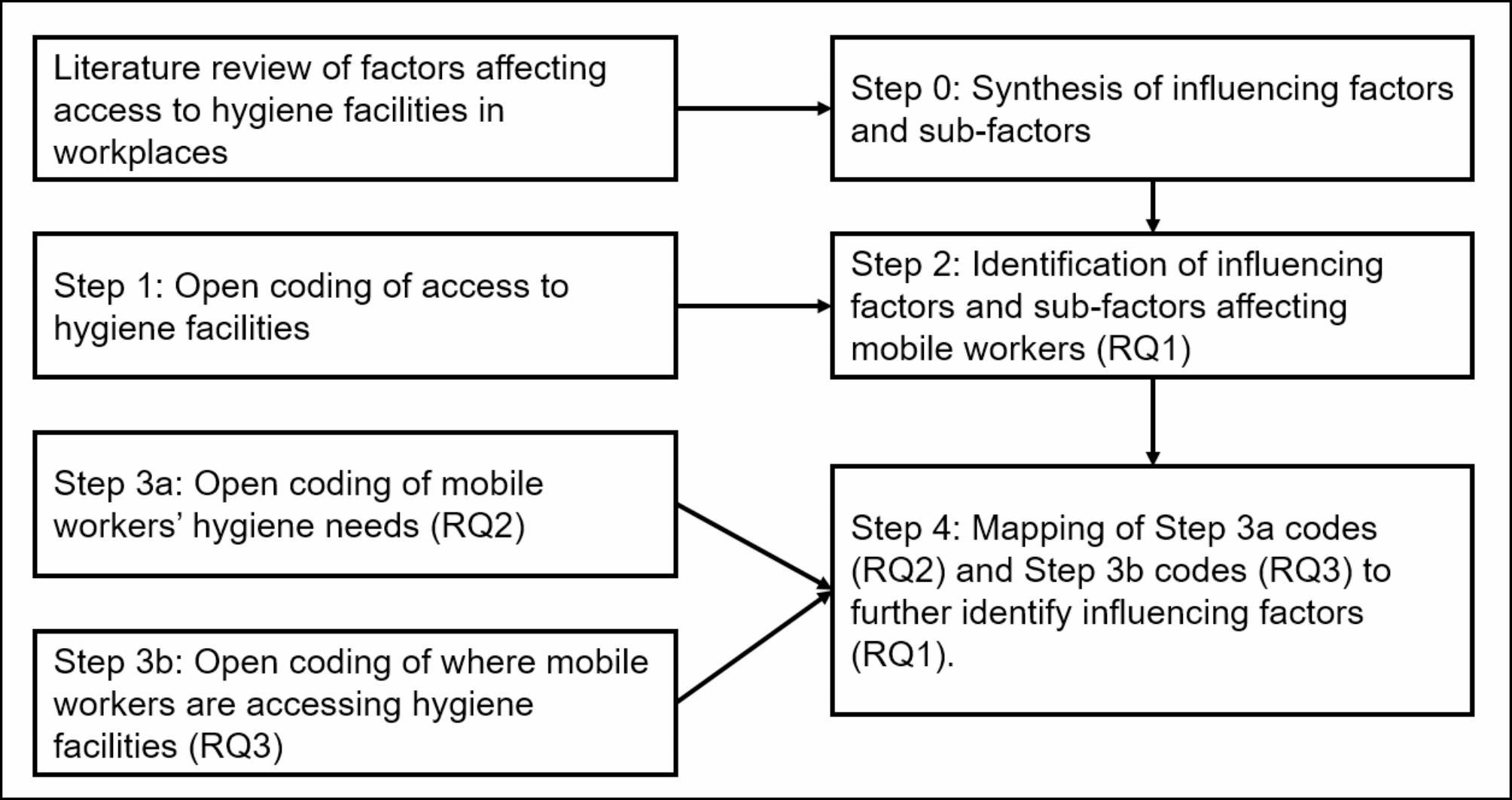
Data analysis steps

A “codebook” approach to thematic analysis [51] was taken whereby a structured coding framework was developed deductively from a review of the literature with new codes added inductively and reflexively during the analyses of the interview transcripts. As is typical with this approach inter-rater reliability was not measured [51]. To mitigate against any potential biases, all authors participated in the data analysis process; codes were discussed and agreed upon. The data sets were manually coded in NVivo 12 with the unit of coding being the full turns of speech. The data was analysed in 4 steps (see Fig. 1) by the study authors (SR, AM & LW).

- In step 0, SR synthesised the literature (see Supplementary Material for a summary of each study reviewed) on access to hygiene facilities discussed in Sect. 1.3 to identify factors known to influence hygiene within fixed workplace settings.
- In step 1, to capture *all* factors that might be influencing mobile workers’ access to hygiene facilities, preliminary open coding was carried out by AM, LW and SR with each analysing two transcripts. At a follow-up meeting, developing codes were discussed and compiled. AM completed the coding of the remaining transcripts.
- In step 2, SR reviewed and mapped the mobile workers’ codes to factors identified in fixed workplace settings (step 0) while also recording newly identified factors.
- In steps 3a and 3b, SR identified mobile workers’ hygiene needs (RQ2) and where they are accessing hygiene facilities (RQ3).
- Then in step 4, SR mapped these step 3 codes to the factors from step 2 to further identify what factors influence access to hygiene (RQ1).

## Results

### What is influencing mobile workers’ access to hygiene (RQ1)?

From a review of the literature (see Supplementary Materials), we identified the wider cultural environment, the biological environment, the organisational environment, the worker’s role and the physical environment as factors influencing access to hygiene in fixed workplaces, with social norms underpinning all factors. During our interviews, mobile workers described these factors too, with some differences in the sub-factors. Our participants also reported an additional factor of facility owner. We report these results in Table 1 and in more detail next.

For the organisational environment, our participants did not report the influence of leadership, and only one reported receiving information and support from their organisation on finding and accessing toilet facilities. Our participants only received hand hygiene training if it was deemed important to their role (for example, if their work involved providing care). Some of our participants did, however, report informal information exchanges, a sub-factor that was not identified in the literature review for fixed workplaces. Whether or not information exchanges occur, seems to depend on the nature of the profession, and whether there is team work

“Sadly, mobile hairdressers don’t seem to be friends with other mobile hairdressers because you’re on their patch. No, that’s the sad thing, there was no sort of community to sort of have a discussion about this, you know, it’s just quite cloak and dagger for some reason.” (Mobile hairdresser).

“You’re new kid on the block when you’re training and so they tend to, these are the types of things you’d have in a briefing or after a briefing, or in a coffee room. It’d be common knowledge by the time you’d been a probationary you’d know exactly where you can call, you learn all the hoops.” (Police woman).

When considering the physical environment, safety and appropriateness were reported as additional sub-factors. This is because seasonal workers and those who work outside standard opening hours did not always feel safe visiting public facilities. The appropriateness of the setting in which the hygiene facility is located was also a concern for some occupations.

“When we’re on duty we’re not supposed to go into licensed premises in uniform, unless we’re dealing with an incident.” (Community policeman).

Amongst individual factors, our participants also described bodily needs (“Sometimes I’ve been absolutely bursting” - Community care worker #2). This is likely to be a need for all workers but was not identified in the literature review.

As mobile workers often need to access hygiene facilities outside of the aegis of their employer, facility owner is newly identified as an influencing factor. Our participants reported that their relationship with facility owners depends on the (written and unwritten) policies and rules of the place they are visiting, which can also be influenced by national polices and the biological environment.

“We don’t go in the house because our boots have got grass and stuff on them, so we have to stay in the garden.” (Gardener #2).

“When I started at [Name of charity], because of the nature of their work and the nature of the kind of service users being more at risk, they were saying that you have to have a test before you come in, you have to wear a mask moving around the building.” (Archivist).

To access facilities mobile workers must often seek permission. Whether workers felt able to do this often depended on the relationship between the worker and the place they were visiting, which was influenced by the length of the relationship and how the profession is viewed by the facility owner.

“I’ve got to have been going to see them for a bit before I’ll even ask to go to the toilet. Like on the first day, I don’t go, “Can I go and use your toilet?” I wouldn’t do that.” (Community care worker #3).

“[Wearing a uniform helps] because people trust you. So you know if I were at somebody’s house and I said, ‘Oh can I, you know?’ Or a Police Officer came in, ‘Can I use your toilet?’ ‘Yeah, of course you can, it’s upstairs.’” (Community policeman).

**Table 1:** Factors influencing access to hygiene in fixed and mobile workplaces

Influencing factors	Sub-factors
Social norms (underpins all other factors)	
Wider cultural environment	● Social ● Political
Biological environment	● Visibility and presence of disease, faeces and urine.
Organisational environment	● Organisational ownership & structure ● Domain of interest & organisational culture ● Policies, regulations, rules & encouragement ● Leadership*** ● Training & education*** ● Informal information exchange*
Facility owner*	● Policies / guidance / regs* ● Relationship with worker* ● View of the visiting profession*
Worker’s role(s)	● Workload ● Workflows & procedures ● Professional status ● Work tasks and competing responsibilities ● Working hours
Physical environment	● Location of ● Accessibility of ● Visibility of ● Cost to use ● Privacy of (individual or communal) ● Access to hygiene materials ● Quality of facilities and hygiene materials ● Safety of* ● Appropriateness*
The individual	● Bodily needs** ● Knowledge ● Behavioural capacity ● Motivation ● Habits ● Planning

*Newly identified for mobile workers

**Identified in this study but likely an issue for all workers. (Note, the identification of factors influencing access to hygiene in fixed workplaces was based on a review of hand hygiene behaviour (Sect. 1.3) rather than the need to access toilets)

***Little evidence of for mobile workers

### What are the hygiene needs of mobile workers (RQ2)?

Mobile workers need hygiene facilities: so they can use the toilet; to clean their hands, body and clothing; to do their work; and for First Aid (Table 2).

Our participants reported that they may be working some distance away from their workplace or home facilities. A key reason for them needing to access hygiene facilities is so that they can use the toilet. Older workers, workers who are menstruating, and workers with health conditions were particularly adversely affected.

“I could be out and about for several hours not near a toilet.” (Park ranger).

“I’m coeliac, so if I ever eat wheat, I do need to use the toilet pretty quickly.” (Child & youth support worker).

Participants also sought hygiene facilities to clean hands, bodies and clothing prior to handling food; or to clean up after coming into contact with body fluids, chemicals, dust, grime, spilled food, or other, non-specified forms of tangible or visible dirt. Hands were also cleaned to remove germs before and after coming into contact with people and items that are frequently touched. The need to clean hands was influenced by fear of contracting COVID-19 and other infections, with some mobile workers more likely to come into contact with visible dirt and germs through their professional activities (such as police, care workers, gardeners).

“[A friend’s] dad was a window cleaner, and he was ill for a week, and it turned out it was a bug from bird mess that he’d got on his hands.” (Window cleaner).

Hand hygiene is also influenced by an individual’s habit, motivation and social norms.

“If I use a public facility, I would always wash my hands, even if I’m going to pee. And I know there are lots of men that don’t do that, I always do that. But if I’m out and about, yeah, I almost see it as an opportunity to rebalance from all the scrubbing that we do in a more urban environment.“ (Park ranger).

Mobile workers reported needing hygiene facilities for their work. Healthcare workers reported that they are required to clean hands before, during and after contact with a patient. When community workers took those they are looking after out on trips they needed to support them with finding and sometimes using facilities. Other workers reported needing hygiene facilities to clean their tools.

“He [a child] just, basically, threw up down himself …we were in a café, I’m just like, ‘Get a wipe then and wipe it off’”. (Child & youth support worker)

“Clean my dishes, clean my brushes.” (Mobile hairdresser).

One participant who worked some distance away from facilities was also concerned about accessing First Aid.

“The First Aid angle, you know, if you’re out and about remotely and you cut yourself or something like that, you need to make sure that the wounds are clean.” (Park ranger).

**Table 2:** Mobile workers’ hygiene needs and influencing factors

Hygiene needs of mobile workers	Influencing factors
Needing the toilet	● Worker’s role: Workload, working hours and work tasks ● Physical environment: Location of and accessibility of facilities ● Individual bodily needs
To clean hands, bodies and clothing	● Biological environment: Exposure to dirt and germs ● Organisational environment: Domain and policies ● Physical environment: Location of and accessibility of facilities ● Worker’s role: Work tasks ● The individual: Motivation and habit ● Social norms
For their work	● Wider environment: National and international hygiene policies ● Biological environment: Exposure to dirt and germs ● Organisational environment: Domain and policies ● Physical environment: Location of and accessibility of facilities ● Worker’s role: Work tasks
For First Aid	● Physical environment: Location of and accessibility of facilities ● Worker’s role: Work tasks

### Where are mobile workers accessing hygiene facilities (RQ3)?

Mobile workers may access hygiene facilities in the place they are working, they may find hygiene facilities while they are travelling, and/or they may carry hygiene kits. Sometimes, they are unable to access hygiene facilities.

### Accessing facilities where they are working

Our participants reported using hygiene facilities (if available) in the places where their work took them. These could include facilities inside and outside of: people’s homes, a host organisation, and places visited as part of their work (such as parks and other public places).

If their role allowed it, our participants planned their days around where and when they could access hygiene facilities. When planning toilet breaks they also took into consideration whether accessing particular facilities might mean taking time away from work and with it a potential loss of earnings.

“I could ask either one if I could use their toilet. One… I can go in the toilet, wash my hands, in and out, the other one I’d be in for 30 minutes because they’d want to have a chat.” (Window cleaner).

When a mobile worker is responsible for other people, it can make it even more difficult to access facilities.

“The one I mainly look after, who I take out … and if I want to go to toilet I do have to go and say to her, “Are you OK while I go to toilet?” If she says yeah, then I’ll go, but if she says, “Not at the moment,” I do have to wait”. (Community care worker #3)

A common concern was the quality of hygiene facilities in homes, commercial premises and public places, including facilities that smell bad; lack toilet paper, hot water, soap, sanitiser, hand wipes; and where the soap, towels, toilets and door handles are dirty. Participants also reported a preference for some types of hygiene facilities and materials. For example, pump dispensers over bars of soap, and paper towels over hand dryers. For the mobile hairdresser, who needed to clean tools, sinks that automatically dispense soap, water and hot air were a particular problem.

“You get a quick three, four second wash, blob of soap, then a hand drier, so yes, you have to keep doing it again and again . and apologise to anyone in the queue.” (Mobile hairdresser).

A further concern for our participants was the lack of privacy that occurs when using communal facilities and using facilities while being responsible for another person. This can also be affected by the policies of the facility owner, such as whether visitors need to be accompanied.

“[On needing to share a cubicle in a coffee shop] I’ve known her for a very long time, she was very good and discreet as I was as well in turning backs, and stuff like that. She was very good.” (Community care worker #2).

“And in schools, they have to wait around for you – they have to stand outside the door for safeguarding reasons. So you’d sometimes be sat in one cubicle while someone’s stood in the toilet, like, “Please, go away.” (Travelling IT Salesman).

### Travelling to find facilities

Our participants reported travelling to find hygiene facilities, including in commercial premises (such as supermarkets, petrol stations, fast-food restaurants, pubs, garden centres, tea-rooms and coffee shops), public premises (such as libraries, churches, council buildings and civic centres) and public toilets. They may also temporarily return to their homes or work bases. While they are travelling to find hygiene facilities our participants reported concerns about quality and privacy similar to those reported when accessing hygiene facilities in places where they are working. We focus next on additional concerns.

When travelling, our participants reported needing to find facilities en-route, or to make detours. This is challenging as there has been a decline in the provision of public toilets, and with many department stores and pubs closing there are fewer accessible facilities. This was particularly apparent in rural areas.

“Sometimes with the odd garden centre that has a restaurant and loos there …, it’s not that easy once you go out into the wilds of the English countryside.” (Travel photographer).

The type of organisation our participants worked for and the nature of their employment contract affected their ability to travel to facilities. Some employers factored hygiene-related breaks into the working day but for hourly paid workers travelling to a hygiene facility meant they risked not completing their work. Furthermore, the additional travel costs money.

“You leave your route, you’re wasting 20 minutes or 30 minutes to go to the toilet.” (Delivery driver #2).

“Because I’ve got to think about the petrol as well.” (Community care worker #2).

Few of those interviewed said they felt comfortable asking for permission to use facilities in commercial premises, and only a few were aware of, or used, card schemes^1^ which can provide free access to hygiene facilities. Our participants valued supermarkets and other places where they felt they could go without spending their earnings purchasing items and without taking time away from work to consume any food or drink purchased.

“I would normally tend to go to [named fast food café]. But then you’re spending what bit you’re earning, you’re spending on, you know, having a drink or something like that. But you know, I mean when I’ve got that time, if I do, it’s only cheap in there anyway, so. But yeah, and use their toilets.” (Community care worker #1).

It can also take time (and therefore time away from work) to find facilities. To help them find the closest or most convenient toilet, workers are reliant on their knowledge of areas.

“I think the main problem, problem one, is knowledge of the area that you’re in. So I went to the next town on the other week with a kid, and if I’d have needed to go to the toilet there, I’ve got no idea where I’d have gone – I’d have been scratching around for one. So local knowledge is paramount of what’s possibly available and what actually is available.” (Child & youth support worker).

### Carrying their own hygiene kits

Some of our participants reported that they carried their own hygiene kits because they were often not able to access hygiene facilities. Employing organisations may provide resources (such as gloves and sanitiser) and temporary facilities (such as portaloos and welfare vans). Many mobile workers received slightly more support when the restrictions associated with COVID-19 were in place:

“…when there was the COVID regulations, they [organisation] were giving, on a regular basis, the disinfectant and the wipes, so we had a little collection of disinfectant at home, and now we are just using that.” (Delivery driver #1).

Many of our participants purchase their own hand sanitiser and wipes. They may also purchase gloves to avoid the problem of dirty hands, and many have other innovative solutions to overcome access problems.

“…built in the back of the van, a tap on a pump, a 12 hour pump. So basically turn the tap, just a normal tap, but it has a micro switch on it and it turns, then basically I had my own running water in the back of the van…” (Heating engineer).

“…use the wrapper from the sandwich to hold the sandwich while I ate it.” (Sound engineer).

The worker’s domain, competing priorities, personal attitudes and social norms influenced purchase and use of hand hygiene kits.

“Weight is an important aspect for people who are not in the Air Force, particularly if they’re going to be away for a long period. And going for a four-day patrol through the jungle, you are not easily re-suppliable. And in fact you would throw a lot of stuff away.” (Military officer).

“I’m going nowhere without a tissue, and I’m more aware of touching door handles, very reluctant to go to public toilets unless I have to, avoiding handshakes, using my elbow, or greeting new people even though it’s something that I try to negotiate with new contacts.” (Food courier).

### Taking alternative actions

Our participant reported that when they are unable to access facilities they may restrict what they eat and drink, ignore urges, go to the toilet outdoors and in extreme situations soil their clothes.

“It’s not healthy really but you try to limit what you drink so that you don’t actually need the toilet.” (Window cleaner).

“I waited a long time until we found a place where it was just trees and no houses around, and I just went behind the van basically.”(Delivery driver #1).

“Before I actually went to the toilet I actually weed myself [laughs]. Oh god, that’s how bad I can get.” (Community care worker #3).

## Discussion

Access to hygiene facilities is challenging for mobile workers, and is influenced by a broad range of factors including the wider cultural environment, the biological environment, the organisational environment, the facility owner, the worker’s role, the physical environment and the individual themselves, with social norms underpinning all factors (RQ1). Our study also found that mobile workers need hygiene facilities so they can use the toilet; to clean their hands, body and clothing; to do their work; and for First Aid (RQ2). Unlike those working in fixed workplace settings [9] there is often little or no provision for mobile workers to access hygiene facilities while they are at work. Instead, our study finds that when mobile workers need to access hygiene facilities they must seek permission to use them where they are working, travel to hygiene facilities or use hygiene kits (RQ3). In the absence of facilities, workers often have to resort to strategies that may affect their health (such as restricting drinking and eating, and ignoring urges) or their dignity (such as going outdoors or even soiling their clothes). This is an important public health concern.

### Barriers to accessing hygiene facilities increase with being mobile

The barriers in accessing hygiene facilities faced by workers in fixed workplaces are also experienced by mobile workers, namely the wider cultural environment, the biological environment, the organisational environment, the physical environment, the worker’s role, and the individual themselves (Table 1). In addition, we identified new factors influencing access to facilities that have not been identified in previous studies of workplaces (also Table 1) including: facility owner as a factor; safety and appropriateness of facilities as sub-factors in the physical environment; and informal information exchange as a sub-factor in organisational environment. We believe that these new barriers are directly related to mobile workers needing to access facilities outside of a fixed work base. Firstly, mobile workers need to access facilities in public, private and commercial places. This access is dependent on the facilities being available, a relationship with the facility owner, the facility owner’s policies and their view of the profession. Secondly, mobile workers need to access facilities that are not under the aegis of their employing organisation, yet for some workers (e.g. police, those working with children) their professional standing means that they are restricted from entering certain premises thought inappropriate. Thirdly, again because the facilities are not under the aegis of their employer, mobile workers may not feel safe going into some facilities. This, of course, relates to inequalities around public toilet access discussed in Background. Finally, it is likely the social isolation and reduced access to resources experienced by mobile workers [12] are prompting informal information exchange.

### Lack of facilities affects mobile workers’ health and well-being

Mobile workers are not always able to gain access to hygiene facilities. This could damage the health and well-being of mobile workers, those they look after and the wider community. Firstly, mobile workers need access to facilities to clean their hands, body and clothing. Not doing so means they are at greater risk of contracting and spreading gastrointestinal and respiratory infections [52, 53]. Secondly, to avoid needing to use the toilet, mobile workers are restricting what they eat and drink. This puts them at risk of constipation and with it haemorrhoids, chronic pain and urinary tract infection [5]. When mobile workers need the toilet and are unable to access facilities they run the risk of soiling their clothing and/or urinary tract infections associated with infrequent voiding [4]. Thirdly, many of those in our study found it uncomfortable to ask for access, and having to declare need can feel disruptive to a person’s privacy [54]. Furthermore, being denied access is stressful and can lead to feeling a loss of dignity [55]. Fourthly, mobile workers need access to facilities to carry out their work. Some mobile workers need access to facilities for their tools. Mobile workers in caring professions need access to facilities to care for those they support. Lack of hygiene facilities limits where mobile workers can take those they support.

### Quality of facilities is a concern for mobile workers

The UK HSE advises that “you must also ensure that the facilities are kept clean and in good condition, and that there is always an adequate supply of toilet paper, soap, etc” [20]. For the mobile workers in our study, quality of facilities is a major concern, with participants reporting a host of problems including dirty facilities and a lack of materials. Furthermore, there are privacy concerns in using communal spaces, particularly if the mobile worker is caring for another person and cannot leave them unattended.

### Accessing hygiene facilities costs mobile workers

To access commercial and public facilities, mobile workers may need to pay an entrance fee or purchase goods (such as tea and coffee). Workers may suffer further financial loss if they need to take time away from work to travel to facilities. This is further impacted when access intersects with the pressures of (often insecure) employment demands and pay is on the line. Finding facilities is increasingly difficult as many public toilets and department stores with toilets have closed [56]. This means that when their job allows, mobile workers plan their days, deciding where they go and in what order so that they can access hygiene facilities.

Many of the mobile workers in this study reported needing to purchase their own hygiene kits (e.g. sanitiser, wipes, gloves) because they might not be able to access any hygiene facilities or the quality of the facilities is inadequate (e.g. no soap). Some organisations (particularly those in the health domain) supply sanitiser and other forms of PPE that can help protect mobile workers from contracting and spreading infections. Unions have also done vital work to improve access to PPE for workers from a range of occupations to protect them from COVID-19 [23]. Nonetheless, that some mobile workers still need to purchase their own kits is inequitable. Furthermore, the cost of supplying such kits might impact on the willingness of employing organisations to provide them. If employing organisations do not prioritise hygiene, they may also be unsure as to whether workers would use the kits.

### Mobile workers need more support and information

Mobile workers in our study took innovative approaches to address hygiene problems (such as building sinks in vans) and were knowledgeable about hygiene facilities in their areas. However, accessing facilities is particularly difficult if a worker needs to visit a new area and/or does not have a personal relationship with facility owners. Working away from a centralised base means that mobile workers are often isolated and lack resources [12]. While some organisations (particularly in the health domain) train mobile workers in hygiene practices (for example using the World Health Organisation’s ‘Your 5 Moments for Hand Hygiene’ [57]), our participants reported that there is very little, or no training and information about where mobile workers can access hygiene facilities or how to negotiate access. Camaraderie among peers may mean that there is informal information exchange but this is dependent on the occupation, and where workers are competing for work, information may not be shared.

### Recommendations

The following recommendations are made to policy makers and organizations to address the inequalities in access to hygiene facilities for mobile workers, based on the findings of the study.

- Recommendation 1: Workplace hygiene regulations should be updated to support and enable mobile workers’ access to hygiene facilities.
- Recommendation 2: Workplace hygiene regulations should be amended to allow all workers to take toilet breaks as necessary, including when mobile.
- Recommendation 3: Organisations should arrange and promote designated rest stops for mobile workers.
- Recommendation 4: Local authorities should be obliged to plan for and provide hygiene facilities, including toilets and washing facilities. These facilities must be kept clean and have an adequate supply of materials such as toilet paper and soap.
- Recommendation 5: Mobile workers should be able to access public hygiene facilities without any financial cost to themselves.
- Recommendation 6: Toilet card access schemes should be further developed to encourage and incentivise commercial establishments to make their hygiene facilities available to mobile workers.
- Recommendation 7: Employers need to make sure they comply with workplace hygiene regulations in providing portable and mobile facilities. Such equipment should be easily obtainable and affordable for mobile workers who are self-employed.
- Recommendation 8: Hygiene kits including sanitiser should be freely provided to mobile workers as part of their employment contract. Provision should be easily obtainable and affordable for mobile workers who are self-employed.
- Recommendation 9: Organisations should provide mobile workers with training and information resources that support them with access to hygiene facilities.
- Recommendation 10: National and local authorities should develop outreach and education programs for all mobile workers, including those from marginalised and/or underrepresented communities.

### Limitations and future work

The sample size of 22 UK mobile workers, while providing some insights, is not representative of the entire mobile worker population in the UK. Moreover, there is likely a selection bias given that many of our participants were recruited using snowball sampling and social media. However, we recruited mobile workers from diverse occupations where either movement is essential for the work (e.g. delivery drivers) or where movement is necessary if work is geographically displaced (e.g. carers) [11], and therefore were able to capture a range of views. A larger and more diverse sample would enhance the generalisability of the findings.

We did not recruit business travellers nor migrant workers. Future research could usefully investigate hygiene barriers of these mobile workers, and consider intersections of inequality, for example migrant workers working in multi/cross cultural contexts and geographical locations. Furthermore, a survey could identify the extent to which mobile workers from different occupations and backgrounds are able to access hygiene facilities, as well as the impact on mobile workers’ health and well-being, and the wider community they serve. This could help identify the scale of the problem, which occupations and which individuals are affected, and how.

While the focus of our study was on the experiences of mobile workers, it would be helpful to investigate the organisational, governmental and regulatory perspectives to consider who has responsibility for providing access to hygiene facilities and how this can be delivered.

## Conclusion

This study reveals the everyday challenges faced by mobile workers trying to access hygiene facilities while they are working. Our participants reported that it is increasingly difficult to access public and commercial facilities, the quality of facilities is frequently poor, workers must often seek permission to use them, or may incur financial costs to access facilities, by having to spend money and/or needing to take time away from paid work. Our participants often relied on the goodwill of people in private homes, and many needed to buy their own hygiene kits and develop innovative workarounds. When they are unable to access facilities, our participants reported restricting what they eat and drink, ignoring urges, going outdoors and soiling their clothing. This is a serious health and well-being concern for mobile workers and communities as a whole, and yet mobile workers’ access to hygiene facilities and the health implications has been little investigated.

By interviewing workers across several occupations, the findings of our study indicate the wide range of people affected: delivery drivers, the military, the police, cleaners, community workers, utility workers, construction workers and so on. The diverse ways in which work is mobile, and therefore the scale of the problem, needs to be recognised.

This study adds to our understanding of hygiene in workplaces, and highlights the inadequacy of current legislation that appears to be primarily serving those working in fixed workplaces such as offices [9]. By considering mobile workers, it also adds to our understanding of inequalities of access to hygiene facilities in the wider community. Furthermore, this study adds to our understanding of the demands of being mobile for work, the hygiene needs of mobile workers, and how being on the move influences mobile workers ability to access hygiene facilities, and the impact this has on their health and well-being [10, 13–16]. We contribute a framework for analysing what is influencing access to hygiene facilities in fixed and mobile work places, which can be used by, policy makers, organisations that employ mobile workers and public health researchers to identify and address access concerns for different occupations. We also make recommendations to policy makers and organisations.

### Electronic supplementary material

Below is the link to the electronic supplementary material.

Supplementary Material 1

## Data Availability

This study involves human research participant data and could contain potentially identifying information. The data that support the findings of this study are available on request from the corresponding author.

## Footnotes

1 There are various card schemes (such as a Just Can’t Wait Toilet Card- www.bladderandbowel.org/help-information/just-cant-wait-card/), whereby a credit-sized card discretely communicates the need for toilet access. These cards are widely accepted in commercial premises.
