# Supplementary material for: Challenges of accessing hygiene facilities when on the move: an exploratory interview study with UK mobile workers
Source: BMC Public Health. 2023 Dec 15;23:2514. doi: 10.1186/s12889-023-17465-y (PMC10722722; doi:10.1186/s12889-023-17465-y)
Supplement: Supplementary file 1 — Supplementary Material 1 [file 12889_2023_17465_MOESM1_ESM.docx]

# Supplementary Material

|  | **Study overview** | **Barriers and enablers** |
| --- | --- | --- |
| Adams et al., 2021 ^49^ | A US study of home health care workers adherence to infection prevention and control practices | - Home environment - Persons - Tasks - Equipment - Health policy environment - Agency environment |
| Appiah-Brempong et al., 2018 ^37^ | A review of hand hygiene research in schools to develop a framework to aid design of school-based interventions. | - Environmental level variables - School regulations (social environment) - Hand washing stations (physical environment) - Individual level variables - Behavioural capacity - Perceived susceptibility - Perceived seriousness - Attitude - Subjective norms |
| Biswas et al., 2019 ^41^ | Hand hygiene compliance in a low-resource neonatal intensive care unit in an Indian hospital | - Structure - Environment - Access to hand wash basins & handrub bottles - Number of hand rub dispensers - Workload - Bed occupancy - Night shift duty - Manpower - Supply - Liquid soap - Hand rub - Paper towel - Process - Barriers to user satisfaction - Too hot / too cold running water - Poor quality of liquid soap & hand rubs - Breach in hand washing steps - No hand drying - Hand recontamination - Administrative - No reminder - No incentive |
| Chittleborough et al., 2012 ^38^ | Factors influencing hand hygiene in UK primary schools | - Structure - Time: Lack of time, competing priorities, routine - Facilities: Attractiveness cleanliness, Accessibility, Soap, Water, Drying facilities - Societal norms: Observing handwashing behaviour - Agency - Encouragement and reminders: - Verbal, - Setting good example, - Visual - Education and information: - School - Home - Awareness & knowledge: - Germs & illness - How to wash hands - When to wash hands |
| Curtis et al., 2009 ^40^ | A study across 11 countries in Asia, Africa and South America of the hand hygiene practices of child carers in domestic settings | - Environmental factors - Physical environment, including the location of facilities, the cost and availability of soap and water - Social environment: the prevailing culture and norms of HH that are promoted through social structures including communities, organisations, governments and mass media. - Biological environment where there might be other bodily priorities, the visibility of disease and the presence of faeces and urine. - Cognitive factors - Planning - Motivation - Habit |
| Green et al., 2007 ^42^ | Food workers hygiene practices (hand washing and glove wearing) in the US | - Worker activity - Activity type - Worker busyness - Hands washed / Gloves worn - Restaurant characteristics - Ownership: chain versus independent, - Com­plex food preparation processes - Worker training - Hand hygiene taught to food workers, - Food safety training provided to food workers, - Management certification required - Physical environment - Multiple hand sinks - Hand sink closeness to worker - Hand sink in worker’s sight, - Hand washing supplies at hand sinks, - Glove supplies in food preparation areas - Social environment and management - Worker visibility to manager - Worker visibility to customer - Man­agement encouragement of hand washing |
| Johansen et al., 2015 ^43^ | The design of a school-based hand hygiene intervention in Denmark | - Structural context - Facilities: Accessibility, Attractiveness - School organisational rules - Individual factors - Attitudes - Subjective norms - Actual behavioural control |
| McMichael & Vally, 2020 ^44^ | Children’s perspectives of water, sanitation and hygiene in schools in the Philippines | - Infrastructure: availability of water and sanitation hardware - Hygiene behaviours - Agency and accountability: perceived responsibilities - Socio-political determinants: the social, economic or political factors beyond the individual student or school context |
| Ramadan et al., 2018 ^45^ | Hygiene barriers among healthcare workers in two paediatric intensive care units in Kuwait | - People - Forgetfulness - Skin dryness - Lack of knowledge - Role model - Policies - Adherence to policies - Procedure - No time - Environment - Lack of leadership - Intensity of patients - Materials - No paper towels - Inconvenient location of hand rub dispensers |
| Shimokura et al., 2006 ^46^ | Factors linked to hand hygiene and glove wearing in US hemodialysis facilities. | - Type of staff - Physician status (vs nurse) - Nursing assistant status (vs nurse) - Male sex - Busyness & type of work environment - Working in an intensive care unit - Working during the week (vs weekend) - Activities with high risk of contamination - High number of opportunities for hand hygiene - Patient to staff ratio - Physical barriers - Irritation and dryness - Sinks inconveniently located - Lack of soap and paper towels - Use of automated sinks - Attitudes - Patient needs take priority - Hand hygiene interferes with health-care worker relationship with patients - Low risk of acquiring infection from patients - Glove use obviates the need for hand hygiene - Scepticism regarding the value of hand hygiene - Knowledge - Lack of knowledge of guidelines and protocols - Lack of scientific information of definitive impact of improved hand hygiene on health care associated infection rates - Institutional factors - Lack of institutional safety climate - Lack of administrative sanction of non-compliers - Lack of active participation in hand hygiene promotion at individual or institutional level |
| Squires et al., 2014 ^48^ | Factors influencing physician hand hygiene in a Canadian hospital | - Relevant domains - Knowledge - Skills - Beliefs about capabilities - Beliefs about consequences - Goals - Memory, attention, and decision processes Environmental context and resources - Social professional role and identity - Social influences - Non-relevant domains - Behavioural regulation - Emotion - Intentions - Optimism - Reinforcement |
| Song et al., 2013 ^47^ | Barriers to hand hygiene in a US hospital | - Supply / Environment - Poor accessibility - No standardised location - Empty dispensers - Skin irritation - No signage - Culture / Behaviour / Attitude - Lack of signs for reminders - Fear of repercussions from speaking up - No accountability - Lack of leadership / Role model support - Denial of hand hygiene data - Knowledge - Lack of knowledge about hygiene policy - Lack of knowledge about compliance rates - Lack of knowledge about appropriate use of hand gel - Lack of understanding the importance of hand hygiene in preventing health acquired infections - Workflow / process - No time to wait for gel to dry - Ability to comply in daily work or special circumstances |
| Watson et al., (2020) ^39^ | Perceived determinants of children’s hand washing in a displaced person camp in Iran | - Environmental barriers - Availability of handwashing materials - Lack of lighting - Conditions (dirtiness, cold water) - Communal facilities - Familial roles - Hygiene promotional exposure - Prescriptive social norms |

Table S1: Literature review summary of barriers and enablers of hygiene in workplace settings
